# Elucidating the underlying components of metacognitive systematic bias in the human dorsolateral prefrontal cortex and inferior parietal cortex

**DOI:** 10.1038/s41598-024-62343-1

**Published:** 2024-05-18

**Authors:** Peiyao Cong, Yiting Long, Xiaojing Zhang, Yanlin Guo, Yingjie Jiang

**Affiliations:** School of Psychology, Northeast Normal University, 5268 Renmin Street, Changchun, Jilin 130024 China

**Keywords:** Metacognition; Systematic bias; fMRI; MVPA, Human behaviour, Psychology, Neuroscience, Cognitive neuroscience

## Abstract

Metacognitive systematic bias impairs human learning efficiency, which is characterized by the inconsistency between predicted and actual memory performance. However, the underlying mechanism of metacognitive systematic bias remains unclear in existing studies. In this study, we utilized judgments of learning task in human participants to compare the neural mechanism difference in metacognitive systematic bias. Participants encoded words in fMRI sessions that would be tested later. Immediately after encoding each item, participants predicted how likely they would remember it. Multivariate analyses on fMRI data demonstrated that working memory and uncertainty decisions are represented in patterns of neural activity in metacognitive systematic bias. The available information participants used led to overestimated bias and underestimated bias. Effective connectivity analyses further indicate that information about the metacognitive systematic bias is represented in the dorsolateral prefrontal cortex and inferior parietal cortex. Different neural patterns were found underlying overestimated bias and underestimated bias. Specifically, connectivity regions with the dorsolateral prefrontal cortex, anterior cingulate cortex, and supramarginal gyrus form overestimated bias, while less regional connectivity forms underestimated bias. These findings provide a mechanistic account for the construction of metacognitive systematic bias.

## Introduction

A fundamental issue in human memory research is the relationship between objective (memory) and subjective (metacognitive monitoring) dimensions of memory. The relationship between objective and subjective memory processes can be studied during learning (memory encoding at study) or retrieval (recall or recognition at test). Subjective or metacognitive monitoring during learning is of particular interest because these processes can enhance learning effectiveness by guiding the allocation of resources at a time when information remains available for learning. As a typical example of metacognitive monitoring, judgments of Learning (JOLs) are individuals' assessments of the likelihood that currently learned items will be successfully retrieved on subsequent tests, usually occurring after learning and before testing^1,2^. A common occurrence in judgments of Learning (JOLs) is that individuals tend to overestimate their ability to recall information learned during the learning phase, yet fail to recall it during a subsequent memory test. This overestimation bias is a significant issue. Conversely, there are instances where individuals underestimate their recall ability, yet perform successfully during the memory test. This is known as the underestimate bias. Numerous behavioral studies have observed this inconsistency between predicted and actual memory performance^3–7^. However, the underlying neural mechanisms remain unclear. This study aims to address this gap by exploring the neural basis of systematic biases in metacognitive monitoring. It emphasizes the inconsistency between memory predictions and actual performance, encompassing both overestimating and underestimating biases. The dual-memory monitoring hypothesis posits that making judgments of learning requires information from both working and episodic memories^8^. Alternatively, the memory strength hypothesis suggests that judgments of learning are based on the strength of working memory^9,10^. The monitoring dual-memories hypothesis and memory strength hypothesis offer partial explanations for systematic biases in metacognitive monitoring. The information stored in working memory is unstable, leading participants to make biased memory predictions based on inaccurate information, thus creating a metacognitive systematic bias. Understanding this metacognitive systematic bias is crucial, as the neural representations of working memory can provide neural evidence for previous theoretical hypotheses.

Neural mechanism research has focused on the underlying neural substrates of metacognitive monitoring, exploring associated brain regions such as the ventromedial prefrontal cortex (vmPFC), dorsomedial prefrontal cortex (dmPFC), and anterior cingulate cortex (ACC)^11–13^. Although researchers have identified the localization of metacognitive monitoring, there is a lack of neural evidence for metacognitive systematic bias. Previous fMRI studies have found that each participant exhibits both underestimated bias and overestimated bias through the relationship between estimate memory performance (JOLs) and actual memory performance. Specifically, high JOLs magnitudes that fail to predict recall represent overestimated biases, while low JOLs magnitudes that accurately predict recall represent underestimated biases^14^. This classification helps to investigate the neural substrates underlying predicted memory outcomes (JOLs) compared to actual memory outcomes (memory itself). This study utilized the fMRI technique, combining univariate analysis methods with multivariate pattern analysis, to observe the neural patterns of systematic bias and clarify the mechanisms of overestimated bias or underestimated bias.

From the perspective of episodic memory, studies have primarily focused on behavioral mechanisms of systematic bias, specifically, overestimating or underestimating episodic memory outcomes in tasks such as color generation, emotion experience, and future events prediction^15–18^. Metacognitive monitoring is a crucial factor influencing episodic memory, playing a pivotal role in understanding the neural mechanisms underlying systematic bias. This knowledge is crucial in enhancing our understanding of efficient learning.

In this study, we employed a classic paradigm of metacognitive monitoring, in which participants encoded word pairs and provided judgments of learning (JOLs). The JOLs paradigm we used featured cue words on the left and a question mark “?” on the right. This setup is believed to prompt retrieval attempts among individuals, as observed in previous behavioral studies on immediate judgments of learning^6,19,20^. Since immediate judgments of learning occur immediately after encoding, essentially involving immediate retrieval attempts, the relevant information remains in working memory. Previous theories highlight that immediate judgments of learning may incorporate working memory information^9,21^. Therefore, it can be inferred that individuals utilize working memory information to formulate their JOLs. Furthermore, the immediate judgments of learning paradigm is similar to working memory paradigms, as both involve judgments made shortly after encoding. Although the neural mechanisms underlying immediate JOLs are not the primary focus of this study, they still need further exploration and investigation. Word pairs have been a frequent choice for metacognitive systematic bias research^3,6,22,23^, often including nouns^22,24^. By selecting word pairs as learning materials, we were able to delve into the neural basis of metacognitive systematic bias. Our research objectives were threefold. First, we aimed to identify the brain regions associated with metacognitive systematic bias. To achieve this, we used univariate analysis to compare neural activation patterns between overestimated and underestimated biases during the JOLs task. Second, we sought to decode the brain regions that encode metacognitive systematic bias. To do so, we employed multivariate pattern analysis to identify brain regions that encoded information about overestimated and underestimated biases. Our third objective was to investigate the neural network of metacognitive systematic bias. The metacognitive network has been studied for over five years^25^. Despite this, there is still limited knowledge about the neural substrates of metacognitive monitoring. It is crucial to utilize effective connectivity analysis to observe neural networks that overestimate or underestimate bias. This neural evidence is significant as it provides valuable insights into the neural substrates associated with metacognitive systematic bias. Specifically, it aids in the construction of a metacognitive brain network that can further our understanding of systematic bias.

## Experiment

### Methods

#### Participants

The sample size in the current study was roughly determined by following previous study using a similar task paradigm^26^. 20 subjects participated in the experiments conducted in the current study. All participants were right-handed, had normal visual acuity or corrected visual acuity, and had no personal or family history of neurological or psychiatric disorders based on their self-report. This experiment was approved by the ethics committee of Northeast Normal University. The present study was in agreement with the Helsinki Declaration and approved by the ethics committee of the Northeast Normal University (Study No. 2022020).The participants signed an informed consent form before the experiment and were paid for completing the experiment and received a payment of 100 CNY once the experiment was completed.

#### Stimuli

The 126 abstract word pairs from Yu, Jiang, and Li (2020) were used, and each item is middle difficulty (0.3 to 0.7) through a memory recognition task^27^. Among them, 120 pairs of words were used for the formal experiment, and the remaining six pairs of abstract words were used for practice.

#### Procedure

**Figure 1:**
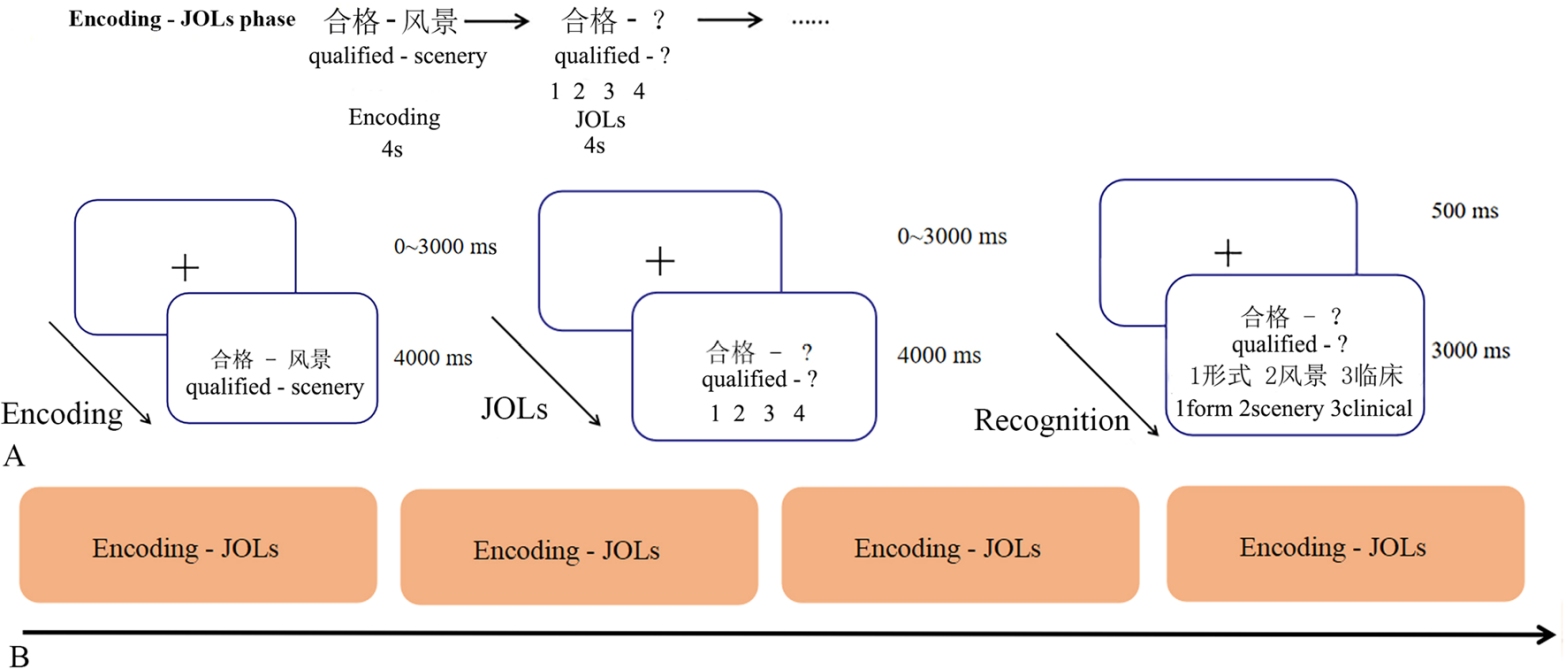
Experiment paradigm. (**A**) The rapid event-related design was used to fit the encoding-JOLs phase better. The major procedure in fMRI contained encoding-JOLs phase. Recognition-test phase was outside the fMRI scanner. Also, the details of each typical trial were introduced. (**B**) The arrangement of scanning runs. There were four encoding-JOLs sessions in total.

In this study, we used an event-related design (Fig. 1). Figure 1 showed the details of the procedure. The formal scan consisted of 4 runs, with a short break given to the participants at the end of each run. The task took approximately 30 min to complete inside the scanner. In the scanner and each run, participants performed an encoding and immediate judgment of learning (JOLs) task. During the encoding and immediate JOLs stage, participants saw random jitters on the center of the screen ranging from 0 to 4000 ms, followed by the presentation of an abstract word pair (e.g., “合格-风景”, written in Latin characters “qualified—scenery”) to be learned for 4000 ms (total 16 word pairs). After encoding each pair, participants saw one word from the pair (the cue) on the screen and were asked to predict how likely they would remember the unseen target in the post-scan recognition task on a four-point scale, with 1 indicating “will be absolutely forgotten” and 4 indicating “will be absolutely remembered”. Participants had 4000 ms to press a button to indicate their estimated performance, and responses were collected online using an MRI-compatible button box. After the encoding-JOLs, a distraction task outside the scanner was asked to complete for 3 min. Participants also were not in the scanner during the recognition-test phase. In a recognition test trial, participants saw a previous cue word that was studied at the top of the screen, and the target word and two distractor words appeared in random locations (left, center, or right) on the bottom. Participants were asked to indicate which of the three words on the bottom had been paired initially with the cue at the top in 3000 ms. Each trial was associated with a fixed-interval fixation of 500 ms.

#### fMRI data acquisition

Neuroimaging data were acquired on a UIH Prisma 3.0 T MRI scanner with a 64-channel head-neck coil. The participant was placed in a supine position with a sponge pad inside the coil to hold the head in place and was asked to keep the head and body still during the scanning process. The functional image was a 32-slice axial image, measured by a T1 -weighted echo-planar images (EPI) sequence, covering the entire cerebral cortex (main technical parameters: TR = 2000 ms, TE = 30 ms, Flip angle = 80°, FOV = 230 mm × 230 mm, Matrix size = 64 × 64, slice thickness = 3.5 mm, sequential acquisition = 32 axial slices, voxel size = 3.5 × 3.5 × 4.2 mm). Each functional scanning session contained 207 time points, with a total of 4 runs. Structural images were collected using a T1-weighted 3D MPRAGE sequence (TR = 7 ms, TE = 3 ms, Flip angle = 9°, FOV = 230 mm × 230 mm, Matrix size = 384 × 384, slice thickness = 1 mm, sequential acquisition = 160 axial slices, voxel size = 0.5 × 0.5 × 0.5 mm), in order to coregister with the functional images.

#### fMRI data preprocessing

Imaging analysis was performed using spm12 (http://www.fil.ion.ucl.ac.uk/spm)^28^. First, all the EPI DICOM data were converted to NIFTI format. The first three images from each run were automatically discarded by the scanner to allow scanner equilibrium. Second, all volume slice scan times were corrected to the middle time slice and realigned to the first scan to correct for head motion. Third, the structural images of each subject were coregistered with the mean functional images, and then the images were normalized to the Montreal Neurological Institute template. Fourth, all voxels were resampled to 3 × 3 × 3 mm. Last, all functional volumes were smoothed by using an 8-mm FWHM isotropic Gaussian kernel.

### Behavioral data analysis

Using participants' responses on the post-scan recognition test, we sorted trials based on JOLs magnitude and recognition performance. At the JOLs stage, participants were required to make immediate JOLs using a 1–4 scale. The 1 and 2 indicate that the participant will forget, while 3 and 4 indicate that the participant will remember. The four-point scale was used to fit the fMRI environment and was based on previous fMRI studies^29^. In the post-scan recognition test, a correct recognition was recorded as 1, and a failed recognition or timeout was recorded as 0. Therefore, items were given either an R (will remember) or an F (will forget) estimation in the JOLs stage and were either subsequently remembered (r) or subsequently forgotten (f) in the post-scan recognition memory test. This study aimed to investigate metacognitive systematic bias by comparing overestimated bias to underestimated bias. The classification of metacognitive systematic bias is of great importance. We classified metacognitive systematic bias into two types^14^: (1) JOLs magnitude was high ("will remember" prediction) but was later failed to recognize in the post-scan recognition test (JOL_high_M_low_), which is overestimated bias. (2) JOLs magnitude was low ("will forget" prediction) but was later correctly recognized in the post-scan recognition test (JOL_low_M_high_), which is underestimated bias. Regarding the classification of the 4-point scale data into two categories, this decision was a deliberate choice, tailored specifically to fulfill the research objectives of elucidating the neural mechanisms that underlie metacognitive biases. By organizing the data into two representative categories, the authors intended to pinpoint and contrast the neural disparities between the two types of metacognitive biases, ultimately disclosing their underlying formation mechanisms. The behavioral data analysis has three steps: First, we calculated response time (RT) and proportion between the JOL_high_M_low_ and JOL_low_M_high_ conditions to test the feasibility of further fMRI analysis. This step was to confirm that both overestimated bias and underestimated bias were not happening by chance. Second, metacognitive sensitivity was calculated for each participant to evaluate the overall metacognitive monitoring accuracy via meta-d/d values in accordance with Maniscalco and Lau (2012). Then, metacognitive sensitivity was calculated for both JOL_high_M_low_ and JOL_low_M_high_ conditions and should be compared between the JOL_high_M_low_ and JOL_low_M_high_ conditions. This approach can provide evidence of which type of metacognitive systematic bias is more sensitive. Because metacognitive sensitivity is an index that measures the accuracy of JOLs^30^. We have known that metacognitive systematic bias has low JOL accuracy, but it remains unclear whether overestimated bias or underestimated bias has less accuracy. Third, the recognition task performance was measured to ensure the effectiveness of the materials and tasks used in the experiment.

### Univariate analysis

The GLM method, as implemented in the SPM toolbox, was used to analyze the BOLD responses to metacognitive systematic bias. For all analysis, events were modeled at the time of the stimulus onset and convolved with the canonical hemodynamic response function (HRF) using a double-gamma function. These events were then superimposed for all trials to fit with the fMRI signals of each voxel. At the JOLs stage, the event was time-locked to the onset of the stimuli, with a duration that was the summation of the presentation period (4 s) and the same duration as the event. The GLM model was based on the JOLs task. The GLM model was based on JOLs task, we separated two task-related events, including JOLs magnitude was high ("will remember" prediction) but was later failed to recognize in post-scan recognition test (JOL_high_M_low_) and JOLs magnitude was low ("will forget" prediction) but was later correct recognized in post-scan recognition test (JOL_low_M_high_). Motion correction parameters were entered as covariates of no interest, along with a constant term per run. The regressors were convolved with a canonical hemodynamic response function. Low-frequency drifts were excluded with a 1/128 Hz high-pass filter. Missed trials were not modeled. We defined two contrasts: JOL_high_M_low_ vs. JOL_low_M_high_ (1 -1), JOL_low_M_high_ vs. JOL_high_M_low_ (− 1 1). Contrasts constructed at the single participant level were then input into a second-level group analysis using a random-effects model. At the group level, metacognitive systematic bias fMRI activation was first obtained by applying a parametric one-sample *t-test*, then a paired sample *t-test* was used to compare the activation between different metacognitive systematic bias (JOL_high_M_low_ versus JOL_low_M_high_, and vice versa). All reported clusters survived a threshold with *p* < 0.05 after correcting for multiple comparisons using the false discovery rate (FDR) method and consisted of ten or more significant voxels.

### Regions of interest (ROI) analysis

ROIs were defined from previous literature ^11,12,14^. Voxels meeting *p* < 0.05 (FDR correction) threshold requirement and lying in the proximity of previously published coordinates of dorsomedial prefrontal cortex (dmPFC) [-6,2,58], ventromedial prefrontal cortex (vmPFC) [− 32,6,54], dorsolateral prefrontal cortex (dlPFC) [-48,24,28], and anterior cingulate cortex (ACC) [0,32,2]^11,12,14^ were taken to be the ROIs used in this study. Beta values were extracted from subjects’ contrast images for the JOL_high_M_low_ and JOL_low_M_high_ univariate analyses, respectively.

### Multivariate pattern analysis

Multivariate pattern analysis (MVPA) was performed in MATLAB using the CoSMoMVPA Toolbox (https://www.cosmomvpa.org/)^31^. According to research on the use of MVPA for decoding in the same field^12^, we classified runwise beta images from GLMs modeling JOL_high_M_low_ and JOL_low_M_high_ activity patterns in ROI and whole-brain searchlight analyses. ROI MVPA was performed on normalized, non smoothed images using the ROI spheres as masks. Previous work has shown that these preprocessing steps have minimal impact on linear discriminant analysis (LDA) classification accuracy while allowing meaningful comparison across subject-specific differences in anatomy, as in standard fMRI analyses^32,33^. A single accuracy value per subject, per condition, and per ROI was extracted and used for group analysis and statistical testing. Whole-brain searchlight analyses used 3 mm-radius spheres centered around a given voxel for all voxels on spatially realigned and slice-time corrected images from each subject to create whole-brain accuracy maps. The significance of the classification accuracies of all voxels was tested using a non-parametric random permutation test (*n* = 5000) and results were corrected for multiple comparisons using the false discovery rate (FDR) approach (the significance threshold was set at *p* < 0.05).

For group-level analyses, these individual searchlight maps were spatially normalized and smoothed using a Gaussian kernel (8 mm FWHM) and entered into one-sample *t*-tests against chance accuracy^34^. Whole-brain cluster inference was performed in the same manner as in univariate analysis. For metacognitive systematic bias classifications, we conducted independent leave-one-run-out cross-validations on JOL_high_M_low_ activity patterns and JOL_low_M_high_ activity patterns. Pattern vectors from three of the four runs in each condition were used to train an LDA to predict the same classes in the vectors from the left-out run. We compared the true labels of the left-out run with the labels predicted by the model and iterated this process for the other run to calculate a mean cross-validated accuracy independently for each condition.

### Effective connectivity analysis

Dynamic Causal Modeling (DCM) is an effective connectivity analysis method for making inferences about causal relationships between brain regions. In this study, DCM was performed in SPM12 to compare brain connectivity strength between JOL_high_M_low_ and JOL_low_M_high_. Specifically, the volumes of interest (VOI) were defined based on brain regions that have significant activation in the univariate analysis and multivariate pattern analysis. In other words, only VOIs were significant in univariate analysis, and multivariate pattern analysis included DCM analysis. Within each VOI, we chose the radius of 8 mm as centers of spherical VOIs based on contrasts within a GLM: JOL_high_M_low_ versus JOL_low_M_high_ and JOL_low_M_high_ versus JOL_high_M_low_. According to previous studies^35^, in DCM analysis, three parameters need to be determined: matrix A (internal parameter), matrix B (modulation parameter), and matrix C (driving input parameter). Matrix A represents the intrinsic coupling among brain regions in the absence of external perturbations, and in this study, matrix A represents the whole metacognitive systematic bias. Matrix B is the change in brain region caused by the experiment, i.e., the JOL_high_M_low_ or JOL_low_M_high_ in this study. Matrix C is the perturbation of brain activity due to external input.

Our primary interest was to estimate the quantitative differences between JOL_high_M_low_ and JOL_low_M_high_ in connectivity strength. Therefore, we focused on quantitative comparisons of the DCM parameters (in particular, matrix *B*) between JOL_high_M_low_ and JOL_low_M_high_. The full model described above was first estimated at the individual level to derive DCM parameters for hypothesis testing at the group level. Then, groups of multiple subjects were averaged using PEB (Parametric Empirical Bayes) and BMR (Bayesian Model Reduction)^35^. The posterior probability (P) > 0.95 was used to indicate the significance of the model. Pairwise tests were also performed between JOL_high_M_low_ and JOL_low_M_high_ conditions, with posterior probabilities (P) > 0.95 indicating the significance of each brain region.

## Results

### Behavioral results

**Figure 2:**
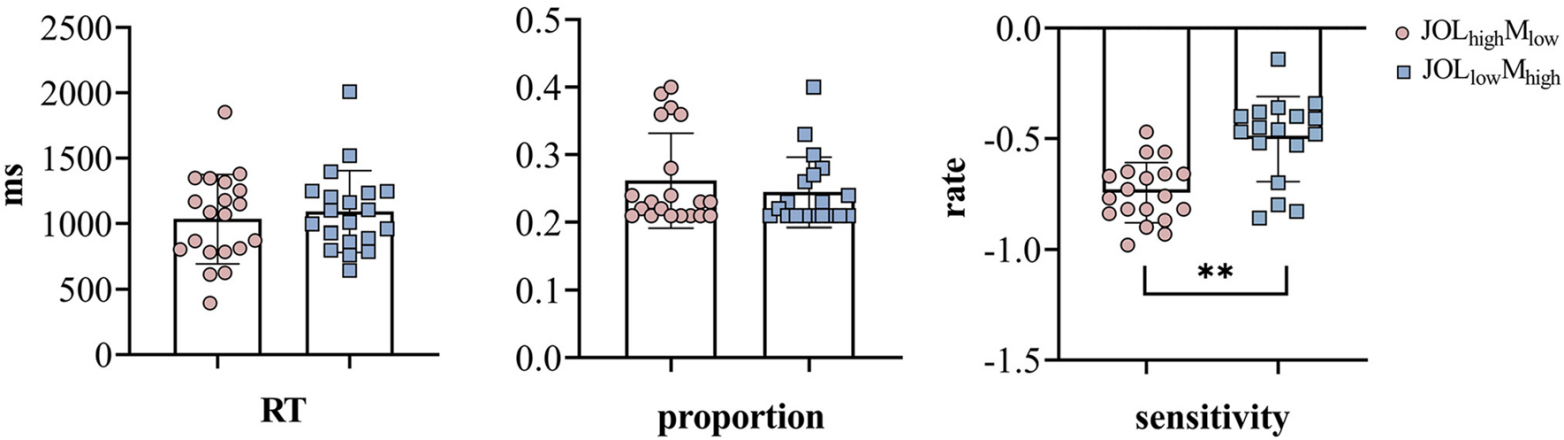
Behavior results in experiment 1. The left panel showed the RT results between JOL_high_M_low_ and JOL_low_M_high_. The right panel represents proportion results and metacognitive sensitivity results between JOL_high_M_low_ and JOL_low_M_high_. **p < 0.01.

Paired sample *t-tests* revealed no significant difference in RT and proportion between JOL_high_M_low_ (*M*_RT_ = 1024.83; *M*_proportion_ = 0.23) and JOL_low_M_high_ (*M*_RT_ = 1169.64; *M*_proportion_ = 0.23), *t*_(16)_ = − 1.75, *p* = 0.099, BF_10_ = 0.876; *t*_(16)_ = 0.039, *p* = 0.969, BF_10_ = 0.249 (see Fig. 2), indicating suitable classification per systematic bias type for further fMRI analysis. Metacognitive sensitivity for each participant was measured via meta-d/d values in accordance with Signal Detection Theory^30^, indicating that participants had lower metacognitive sensitivity, *M* = -1.26 ± 0.23. Then metacognitive sensitivity of JOL_high_M_low_ and JOL_low_M_high_ were measured, and paired sample *t-tests* showed significant difference, *t*_(16)_ = –4.30, *p* < 0.001, BF_10_ = 63.11, means JOL_high_M_low_ have lower metacognitive sensitivity than JOL_low_M_high_. The correct recognition rate for all subjects was 56.60% ± 18%, indicating that the subjects completed the task carefully.

### Univariate analysis results

**Figure 3:**
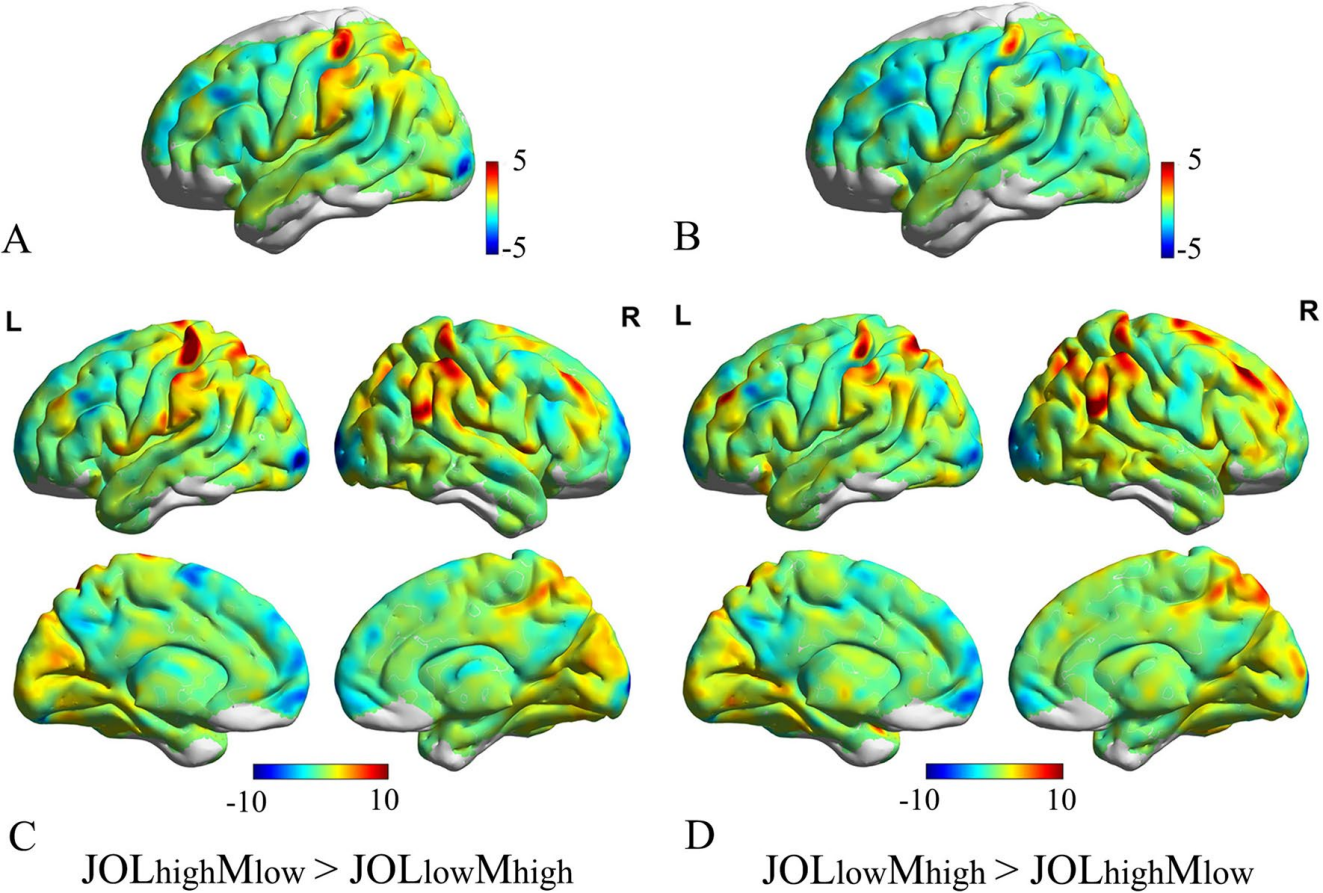
Univariate analysis of metacognitive systematic bias activity in experiment. (**A**) JOL_high_M_low_ activates left dlPFC, left supramarginal, right precuneus, right SFG, left MTG, right STG. (**B**) JOL_low_M_high_ activates left dlPFC, ACC, and left insula. (**C**) univariate BOLD activation in left IPL and left MCC showed a significant difference in JOL_high_M_low_ > JOL_low_M_high_ contrast. (**D**) univariate BOLD activation in the left parahippocampal on JOL_high_M_low_ < JOL_low_M_high_ contrast. *p* < 0.05 FDR correction.

Metacognitive systematic bias whole-brain responses were first analyzed using the conventional GLM method. As shown in Fig. 3A,B, JOL_high_M_low_, and JOL_low_M_high_ all activated left dlPFC and left dmPFC. Other regions activated included left supramarginal, right precuneus, right superior frontal gyrus (SFG), left middle temporal gyrus (MTG), and right superior temporal gyrus (STG) under JOL_high_M_low_ condition. We found elevated activity in ACC and left insula under JOL_low_M_high_ condition (see Fig. 3 and Table 1). Furthermore, comparing metacognitive systematic bias BOLD activation between JOL_high_M_low_ and JOL_low_M_high_ showed other regions activated included left inferior parietal lobule (IPL) and left middle cingulate cortex (MCC) in JOL_high_M_low_ > JOL_low_M_high_ contrast, left parahippocampal in JOL_high_M_low_ < JOL_low_M_high_ contrast (see Fig. 3C,D).

**Table 1:** MNI coordinates and corresponding Z scores for brain areas activated by JOL_high_M_low_ and JOL_high_M_low_ conditions.

contrast	Anatomical Region	MNI coordinates (*x*, *y*, *z*)	*Z* score	*p* value	Hemisphere
JOL_high_M_low_ > JOL_low_M_high_	Inferior parietal lobule	− 52,− 42,56	4.40	*p* < 0.001 *p* = 0.00001	Left
	Middle cingulate cortex	− 4,− 26,36	3.71	*p* < 0.001 *p* = 0.0002	Left
JOL_high_M_low_ < JOL_low_M_high_	Parahippocampal	− 18,− 18,− 24	4.18	*p* < 0.001 *p* = 0.00003	Left
JOL_high_M_low_	Middle cingulate cortex	− 4,− 26,36	3.35	*p* < 0.001 *p* = 0.0008	Left
	Dorsolateral prefrontal cortex	− 48,24,28	6.26	*p* < 0.001 *p* = 0.000000004	Left
	Dorsomedial prefrontal cortex	− 6,2,58	5.15	*p* < 0.001 *p* = 0.0000002	Left
	Supramarginal	− 58,− 24,28	7.09	*p* < 0.001 *p* = 0.00000000001	Left
	Precuneus	10,− 52,28	5.11	*p* < 0.001 *p* = 0.0000003	Right
	Superior frontal gyrus	8,68,14	5.2	*p* < 0.001 *p* = 0.0000002	Right
	Middle temporal gyrus	− 60,− 24,− 14	6.72	*p* < 0.001 *p* = 0.00000000001	Left
	Superior temporal gyrus	48,− 20,6	6.5	*p* < 0.001 *p* = 0.00000000001	Right
JOL_low_M_high_	Dorsolateral prefrontal cortex	− 48,24,28	5.01	*p* < 0.001 *p* = 0.0000005	Left
	Dorsomedial prefrontal cortex	− 6,2,58	4.03	*p* < 0.001 *p* = 0.00005	Left
	Anterior cingulate cortex	0,32,2	5.55	*p* < 0.001 *p* = 0.0000003	Left/Right
	Insula	− 38,− 15,7	3.69	*p* < 0.001 *p* = 0.0002	Left

The ROI analysis results showed no significant difference between JOL_high_M_low_ and JOL_low_M_high_ in left dlPFC (*M* = 0.23, *M* = 0.30), left dmPFC (*M* = 0.40, *M* = 0.35), and left vmPFC (*M* =—0.23, *M* =—0.37). ACC were more activated in JOL_low_M_high_ than JOL_high_M_low_ condition, paired sample *t-tests* : *t*_(17)_ = 4.95, *p* < 0.001.

### Multivariate pattern analysis (MVPA) results

A series of MVPAs were performed to obtain activity patterns of metacognitive systematic bias when remembering abstract word pairs. If systematic bias is shared across JOL_high_M_low_ and JOL_low_M_high_, then common regions would be found in these two kinds of metacognitive systematic bias.

### ROI MVPA analysis results

We performed an LDA decoding analysis using as input vectors the runwise beta images pertaining to JOL_high_M_low_ and JOL_low_M_high_ trials obtained from a GLM (12 input vectors in total). For JOL_high_M_low_/JOL_low_M_high_ classification, we used standard leave-one-out independent cross-validations for each condition (JOL_high_M_low_/JOL_low_M_high_), and we performed one sample *t-test* for each ROI and each condition, then conducted paired *t-test* for JOL_high_M_low_ versus JOL_low_M_high_ to obtain which region decoding metacognitive systematic bias information.

**Figure 4:**
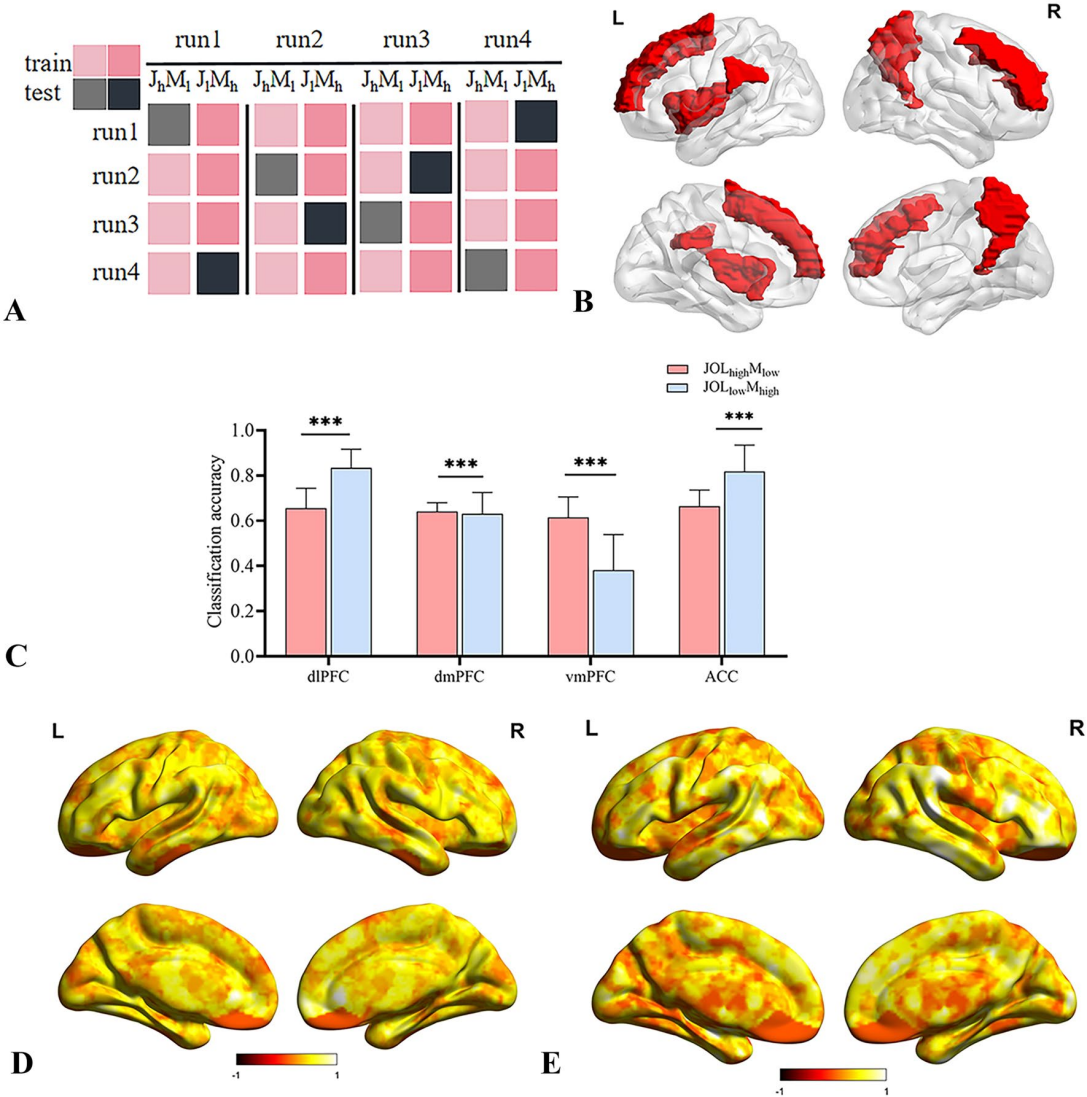
MVPA results. (**A**) Pattern vectors of two classes (e.g., JOL_high_M_low_ and JOL_low_M_high_) were used to train a decoder in a leave-one-run-out design that was then tested in the left-out pair. The process was iterated four times to test pairs from every run. (**B**) Mask used in ROI MVPA analysis (**C**) ROI results for JOL_high_M_low_ versus JOL_low_M_high_ classification accuracy in experiment. (**D**) Searchlight analysis results for JOL_high_M_low_ classification accuracy in experiment. (**E**) Searchlight analysis for JOL_low_M_high_ classification accuracy in experiment. ***p < 0.001. All clusters in D and E are significant at a cluster-based permutation test (*p* < 0.05), corrected for multiple comparisons at *p*_*FDR*_ < 0.05.

Mean accuracy in classifying JOL_high_M_low_ and JOL_low_M_high_ was significantly above chance level in all ROIs (one-sample *t-tests* Bonferroni corrected for multiple comparisons α = 0.05/4 = 0.0125), shown in Fig. 4. In details, the mean accuracy of JOL_high_M_low_ in each ROI: left dlPFC, *t*(16) = 21.68, *p* < 0.001; left dmPFC, *t*(16) = 20.37, *p* < 0.001; left vmPFC, *t*(16) = 4.06, *p* < 0.001; ACC, *t*(16) = 20.66, *p* < 0.001; and JOL_low_M_high_ in each ROI: left dlPFC, *t*(16) = 9.76, *p* < 0.001; left dmPFC, *t*(16) = 7.29, *p* < 0.001; left vmPFC, *t*(16) = 5.12, *p* < 0.001; ACC, *t*(16) = 15.09, *p* < 0.001. In particular, paired *t-test* used to analyze the common regions in ROI analysis showed JOL_high_M_low_ classification accuracy was significantly different from JOL_low_M_high_ in left dlPFC (*t*(16) = 21.68, *p* < 0.001), left dmPFC (*t*(16) = 14.46, *p* < 0.001), left vmPFC (*t*(16) = 5.12, *p* < 0.001), ACC (*t*(16) = 15.09, *p* < 0.001) (see Fig. 4C). Consistent with our hypothesis, JOL_high_M_low_ and JOL_low_M_high_ representations could be decoded in parts of the PFC and temporal cortex.

### Searchlight analysis results

We ran a similar decoding analysis using an exploratory whole-brain searchlight, obtaining a classification accuracy value per voxel when classifying JOL_high_M_low_ and JOL_low_M_high_. As shown in Fig. 4E,F, Consistent with our ROI results, we observed significant accuracy classification under JOL_high_M_low_ condition (Fig. 4E) in left dlPFC (*t*_(16)_ = 15.97, *p* < 0.001), left dmPFC (*t*_(16)_ = 35.74, *p* < 0.001), left vmPFC, (*t*_(17)_ = 14.53, *p* < 0.001), ACC (*t*_(16)_ = 20.06, *p* < 0.001), and significant accuracy classification under JOL_high_M_low_ condition in left dlPFC (*t*_(16)_ = 25.68, *p* < 0.001), left dmPFC (*t*_(16)_ = 14.46, *p* < 0.001), left vmPFC, (*t*_(17)_ = 5.12, *p* < 0.001), ACC (*t*_(16)_ = 15.09, *p* < 0.001) (one-sample *t-test* Bonferroni corrected for multiple comparisons α = 0.05/4 = 0.0125). Searchlight analysis found other regions decoded JOL_high_M_low_ information (Fig. 4F), specifically, left supramarginal gyrus (*t*_(16)_ = 16.33, *p* < 0.001), right precuneus (*t*_(16)_ = 20.26, *p* < 0.001), and other regions decoded JOL_low_M_high_ information: left insula (*t*_(16)_ = 15.14, *p* = 0.000), left IFG (*t*_(16)_ = 10.13, *p* < 0.001), right precuneus (*t*_(16)_ = 11.26, *p* < 0.001). Furthermore, a paired *t-*test was used to analyze the common regions in searchlight analysis and showed higher decoding accuracy for JOL_high_M_low_ than JOL_low_M_high_ in the right precuneus (*t*_(16)_ = 2.73, *p* = 0.016). These results revealed that the different part of the brain region represents information about specific metacognitive systematic bias, and common regions of PFC shared information across JOL_high_M_low_ and JOL_low_M_high_.

### Effective connectivity results

**Figure 5:**
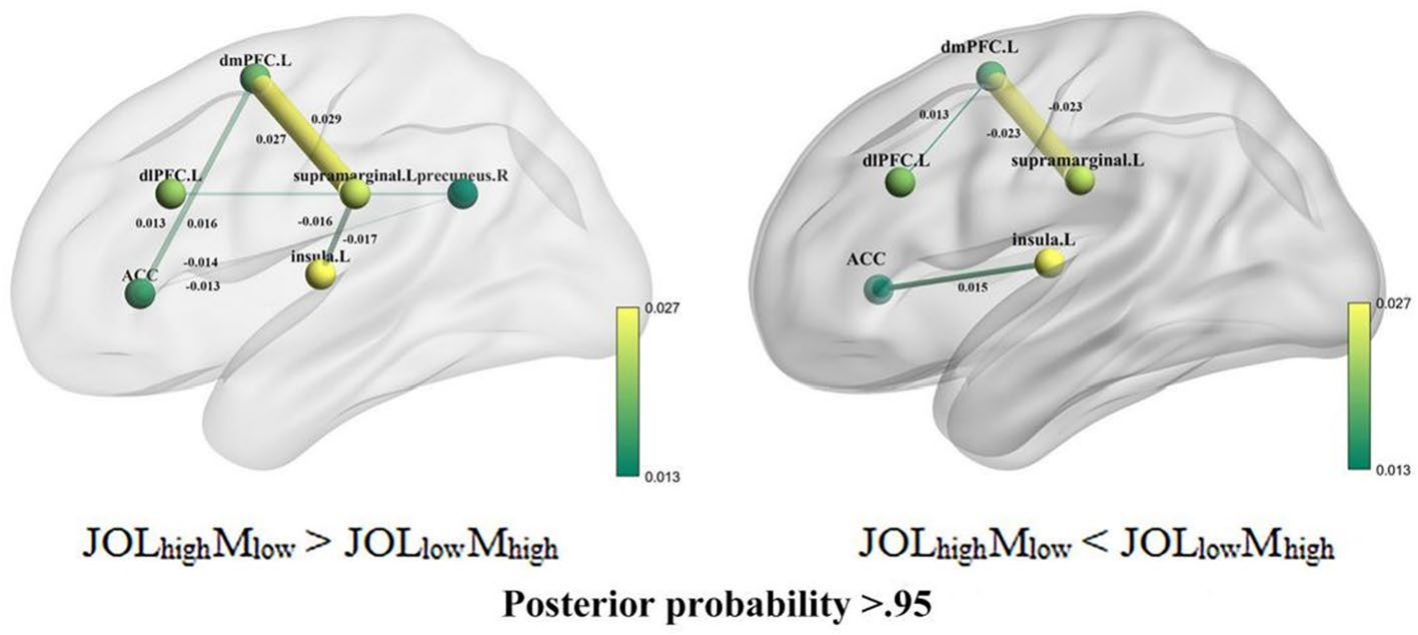
Effective connectivity results in experiment. (**A**) Effective connectivity results for JOL_high_M_low_ > JOL_low_M_high_. (**B**) Effective connectivity results for JOL_low_M_high_ > JOL_high_M_low_. Posterior probability was higher than 0.95 (P > 0.95).

Figure 5A,B shows the PEB analysis results for the modulatory effects on the effective connectivity between the modeled nodes. Connection strengths of the parameters whose posterior probability was higher than 0.95 (P > 0.95) are reported. The results under JOL_high_M_low_ > JOL_low_M_high_ condition found a significant single connection from left dlPFC to right precuneus, and bidirectional connections between left dmPFC and ACC, right precuneus and ACC, left dmPFC and left supramarginal gyrus, left insula and left supramarginal gyrus.

The results under the JOL_low_M_high_ > JOL_high_M_low_ condition showed a significant single connection from left dlPFC to left dmPFC, left insula to ACC, and bidirectional connections between left dmPFC and left supramarginal gyrus (Fig. 5B).

## Discussion

A critical question in metacognitive monitoring is why individuals are sometimes inclined to overestimate or underestimate their memory performances. The neural mechanism of metacognitive systematic bias for overestimate prediction versus underestimate prediction was examined in this study using fMRI, machine learning decoding, and effective connectivity. In particular, we direct our attention on whether metacognitive brain regions and working memory regions engage in the formation of systematic bias when making JOLs. We found dissociated neural mechanisms that supported overestimated bias and underestimated bias, and the results should deepen our understanding of the cognitive and neural mechanisms of metacognitive systematic bias and thus help to answer the question of how this bias occurs.

### Neural correlates of metacognitive systematic *bias*

Our results could help clarify the role of dlPFC in JOLs and the working memory process. As a typical metacognitive monitoring region, the activation of dlPFC was found in previous studies ^13,14,36,37^. One possible explanation posits that increased dlPFC activity reflects partial retrieval of the target word in working memory^14^, but this hypothesis is contradicted by the fact that dlPFC is more dorsal to the regions involved in semantic elaboration^29^. The debate on dlPFC was partly resolved through a TMS study. Rounis et al. (2010) found causal evidence that dlPFC TMS decreases metacognitive accuracy^38^. Using the JOLs paradigm and MVPA analysis, this study found strong evidence that dlPFC represents metacognitive monitoring. Specifically, univariate fMRI analysis showed that the JOL stage evoked metacognitive monitoring-related BOLD activity in dlPFC, and MVPA revealed that the decoding accuracy in dlPFC was significantly above the chance level in the experiment. It is suggested that dlPFC, as a metacognitive monitoring region, plays a fundamental role in the formation of metacognitive systematic bias.

Another region was found in ACC, which is known for performance monitoring^11^, integration of detected conflicts^39^, and attentional control mechanisms^40^. It has been shown that the cingulate cortex plays a major role in detecting discrepancies between the intended and the actual outcome of an action^41^. The significant classification accuracy of the ACC in the context of predicting memory performance (JOLs) might reflect its engagement in general performance monitoring. This result was supported by previous univariate fMRI analysis, and this study observed the ACC through machine learning decoding that supports the basic function of the ACC in the formation of metacognitive systematic bias.

As has been mentioned previously, making metacognitive monitoring predictions requires retrieval of the target word in working memory^9,10,14^. This is because at that time, the slow memory traces are weak, and participants will overestimate or underestimate their memory performance. Some regions represent the storage of working memory, e.g., inferior parietal lobule (IPL), supramarginal gyrus (SMG), angular gyrus, thalamus, superior parietal lobule (SPL)^42–44^. These regions associated with working memory were found in the results of univariate analysis of the experiment. In particular, the decoding accuracy of SMG in MVPA results was significantly above the chance level, which suggested SMG as a region involved in metacognitive systematic bias.

Just as working memory retrieval is an inference in metacognitive monitoring studies^9,10^, partial evidence could support the former hypothesis if working memory representations were found in metacognitive monitoring. This study detected that the working memory representation (SMG) provides critical evidence that making metacognitive monitoring predictions requires information from working memory, giving certain neural mechanism evidence to the dual-memories hypothesis and memory strength hypothesis. Furthermore, SMG not only has a single function for memory monitoring but also works in tandem with other brain regions to predict memory. The working memory trace is a possibility to produce overestimate or underestimate bias. The cognitive and neural mechanisms of overestimate bias and underestimate bias will be discussed in the brain connectivity “Results” section.

Moreover, through searchlight analysis, we discovered an interesting finding: a significantly higher decoding accuracy for JO_Lhigh_M_low_ compared to JOL_low_M_high_ within the right precuneus. This area of the brain, the precuneus, has been recognized as integral to metacognition, as supported by correlational evidence derived from functional activity analyses. For example, previous research has demonstrated a connection between metacognitive performance related to memory decisions and the precuneus^12,45,46^. Furthermore, the precuneus plays a pivotal role in retrospective confidence ratings, exhibiting greater activation when individuals express low confidence^47^. These observations suggest that the activation level of the precuneus serves as an indicator of both high and low confidence ratings. Notably, the present study focused on prospective confidence ratings and discovered that JOL_high_M_low_ decoded more information from the precuneus than JOL_low_M_high_, thereby indicating that the precuneus reflects varying levels of confidence.

Throughout various phases of memory, the precuneus exhibits distinct patterns of activity. Specifically, when individuals provide confidence ratings immediately after encoding (judgments of learning), a stronger activation pattern is observed in the precuneus for higher confidence levels, while a weaker pattern is evident for lower confidence. Conversely, when confidence ratings are made following memory testing (judgments of confidence), a greater degree of precuneus activation is associated with lower confidence levels. Not only does the current study reveal variations in precuneal activity during confidence ratings, but it also suggests that the precuneus serves as the neural foundation for metacognitive biases. Furthermore, it appears that the precuneus contributes differentially to two types of metacognitive biases. In particular, it seems to play a more significant role in overestimation biases compared to underestimation biases, resulting in stronger activation and, consequently, higher decoding accuracy. This result not only corroborates the hypothesis of the involvement of the precuneus in metacognition processes^48,49^, but also strengthens the view of a domain-specificity in the assessment of metacognition^12^.

### The different cognitive mechanisms between overestimated *bias* and underestimated *bias*

Through the formation of overestimate prediction and underestimate prediction, we found that SMG played an important role. However, the behavioral evidence showed that overestimate bias (JOL_high_M_low_) had lower metacognitive accuracy than underestimate bias (JOL_low_M_high_), suggesting different cognitive mechanisms behind them. The effective connectivity analysis results provided a network interpretation of the metacognitive accuracy difference. It revealed that higher brain connectivity was observed between the working memory region (IPL, SMG) and uncertainty signals region (insula) in overestimated prediction. Conversely, elevated metacognitive monitoring connectivity was found in underestimate prediction. A possible explanation for the lower metacognitive accuracy in overestimate bias is that more information increases participants’ confidence^50^. When making judgments of learning, individuals require more resources (e.g., working memory resources and metacognitive monitoring resources). A series of irrelevant information can interrupt an individual’s metacognitive monitoring, leading to overestimated predictions due to inflated memory performance. Conversely, when individuals have limited information, the available resources guide them to make underestimated predictions. The brain connection focuses more on metacognitive monitoring regions, providing neural network evidence for underestimated biases. Previous studies have focused on the behavioral mechanism of metacognitive systematic bias^5–7,51^ and the measurement of bias using the behavioral method. However, they lack direct evidence comparing overestimated bias and underestimated bias. This study provides clear neural evidence regarding the formation of overestimated and underestimated biases and interprets the cognitive mechanism from an information availability perspective.

### Dissociable neural networks supporting metacognitive systematic *bias*

When people are overconfident or underconfident in their memory predictions, dissociable neural connectivity is observed. The effective connectivity results provide evidence that the dlPFC and dmPFC play a central role in metacognitive monitoring processes, as significant connectivity was observed between the dlPFC and SMG, dmPFC, and SMG, especially for overestimate bias. The function of the dlPFC and dmPFC should be discussed in detail. Metacognitive monitoring studies have shown that the dlPFC and dmPFC are key brain regions when making metacognitive monitoring judgments^13,14,29,36,37^, while executive function studies suggest that the dlPFC and dmPFC are involved in working memory processes^52,53^. Using the JOLs paradigm and MVPA analysis, we found that the dlPFC and dmPFC are correlated with metacognitive monitoring, and the SMG represents the working memory process, indicating different neural mechanisms between metacognitive monitoring and working memory. Moreover, connectivity between the PFC and parietal cortex has been implicated in metacognition and decision-making studies^13,36,54^. In studies of decision-making, the ACC, vmPFC, and insula have been found to reveal uncertainty in decision-making^55^. The connectivity between the ACC and vmPFC, as well as the ACC and insula, was found to indicate uncertain decision-making, particularly in cases of underestimated bias, across two experiments. These findings suggest that different neural substrates are involved when making overestimated or underestimated biases. It is proposed that multiple regions, including metacognitive monitoring, working memory, and uncertainty, contribute to the formation of overestimated bias, while the collaboration of uncertainty monitoring and decision-making-related brain connectivity leads to the development of underestimated bias.

## Conclusion

It is concluded that the present study has found a remarkable dissociation between the neural processes that underlie overestimate bias and underestimate bias. The results of MVPA and effective connectivity analyses lend support to the hypothesis that working memory is engaged in metacognitive monitoring, and systematic bias relies on the available information one acquires during the learning process. The different patterns of brain connectivity observed between frontal and parietal regions suggest the formation of distinct metacognitive systematic biases. These findings should enhance our understanding of the neural basis of human metacognitive systematic bias.

## Data Availability

The data sets generated for this study are available on request to the corresponding author.
